# The current distribution of an invasive moss species *Campylopus introflexus* (Leucobryaceae) and models of its future range dynamics in two contrasting climatic scenarios in Europe

**DOI:** 10.3897/BDJ.14.e177294

**Published:** 2026-05-19

**Authors:** Isyaku Abubakar, Jasmina B. Šinžar-Sekulić, Marko S. Sabovljevic, Jovana P. Pantović

**Affiliations:** 1 Institute of Botany and Botanical garden “Jevremovac”, Faculty of Biology, University of Belgrade, Takovska 43, 11000, Belgrade, Serbia Institute of Botany and Botanical garden “Jevremovac”, Faculty of Biology, University of Belgrade, Takovska 43, 11000 Belgrade Serbia; 2 Biotechnology Advanced Research Centre, Sheda Science and Technology Complex, P.M.B. 186, Garki., Abuja, Nigeria Biotechnology Advanced Research Centre, Sheda Science and Technology Complex, P.M.B. 186, Garki. Abuja Nigeria; 3 Center of Plant Biotechnology and Conservation (CPBC), Takovska 43, 11000, Belgrade, Serbia Center of Plant Biotechnology and Conservation (CPBC), Takovska 43, 11000 Belgrade Serbia; 4 Department of Plant Biology, Institute of Biology and Ecology, Faculty of Science, Pavol Jozef Šafárik University in Košice, Mánesova 23, 040 01, Košice, Slovakia Department of Plant Biology, Institute of Biology and Ecology, Faculty of Science, Pavol Jozef Šafárik University in Košice, Mánesova 23, 040 01 Košice Slovakia

**Keywords:** alien species, bryophyte, climate change, global warming, modelling, suitable areas

## Abstract

*Campylopus introflexus* is a suboceanic temperate moss species native to the Southern Hemisphere. It was unintentionally introduced to north-western Europe in the first half of the previous century and it has become invasive. The species continues to expand its European range, spreading eastwards, yet its potential distribution is still poorly understood. In this study, we aimed to identify suitable areas for the potential further spread of this neophytic invasive species across Europe and to assess the impact of climate change on its spread, in order to be considered in flora conservation, protection and management strategies for local bryophytes as well as other low-competitive plant species. We used various species distribution models in two climatic scenarios. These models predicted the current, potential and future ranges of *C. introflexus* in Europe. Based on these models, we identified the main environmental factors affecting its distribution and analysed the changes in suitable habitats under future climatic conditions. Temperature seasonality proved to be the most important environmental factor influencing the distribution of this species. In general, the results suggest that, under the influence of climate change and rising global temperatures, this species will decrease its range size, while the most suitable habitats are expected to shift towards higher latitudes and/or altitudes in the future. These findings support climate as the limiting factor in species expansion, providing a basis for targeted monitoring of the species and key areas in the future.

## Introduction

Biological invasions are an integral part of global change and can have a negative impact on ecosystems and human well-being (Vitousek et al. 1997, Gallien et al. 2010). An invasive alien species is a species that becomes established in natural or semi-natural ecosystems or habitats, is an agent of change and threatens native biological diversity (SSC 2000). Invasive alien species are known to have significant adverse effects on biodiversity and stability and functionality of ecosystem, amongst other things (e.g. Elton 1958, Wittenberg and Cock 2001, Hejda et al. 2009, Vilà et al. 2011). They often lead to a rapid decline or, in some cases, even local extinction of native species, while other threats to biodiversity affect species more slowly (Bellard et al. 2022).

Although invasive alien species are the second largest risk factor for biodiversity loss after habitat destruction (Brennan and Withgott 2005), invasive bryophyte species amongst them have not often been the focus of research on the impact of invasion (Essl et al. 2014). According to Essl and Lambdon (2009), 45 bryophyte species have been identified that are considered alien in at least some parts of Europe. However, only a few of them are appraised to be widespread throughout Europe and pose a serious threat to local biodiversity.

One of these is the heath star moss *Campylopus introflexus* (Hedw.) Brid. (Leucobryaceae, Dicranales), a neophytic moss, native in the temperate Southern Hemisphere: South America, southern Africa, Australia and New Zealand, that became an invasive species in Europe and North America around the middle of twentieth century (Hassel and Söderström 2005). The earliest known discovery in Europe was in the United Kingdom, possibly even in the late 19^th^ century, although its increasing frequency and new localities across England, Wales, Scotland and Ireland were only recorded in the second half of the 20^th^ century (e.g. Richards 1963). Subsequently, it was found in the French region of the Bretagne (Størmer 1958), Italy (Reimers 1956), Belgium (Jacques and Lambinon 1968), Netherlands and Germany (Barkman and Mabelis 1968) and, in the following years and decades, further across many countries of central and northern Europe. It is also likely that this moss came to Europe from several locations at different times (Richards 1963).

The CABI database (Hasse 2022) provides a comprehensive overview of studies on the onset of the species' occurrence in European countries. It shows that *C. introflexus* was widespread throughout Western Europe in the late 1960s and in many Central and Northern European countries in the following decades. Despite it being observed in Latvia and Russia (Kaliningrad Province) in 2000, it was originally recorded in eastern Central Europe (Poland) in the 1980s (Lisowski and Urbanski 1989). Poland has a widely known potential for eastwards expansion (Górski et al. 2016). Numerous countries in southern (Portugal, Spain, Italy), central (Germany, Czechia, Hungary, Poland), eastern (Latvia, Russia) and, more recently, south-eastern Europe have reported findings and spreading of *C. introflexus*.

According to Hodgetts and Lockhart (2020), this species is, so far, known to be present in 39 out of 45 European countries. The first discovered record of this species in south-eastern Europe was made in Slovenia in 2013 (Sabovljevic et al. 2020) and 2014 (Ellis et al. 2014). At nearly the same time, it was found in Croatia by Alegro et al. (2018). In Serbia, *C. introflexus* was firstly discovered in the south-western part of the country, on the Pešter Plateau and is still the only known locality (Sabovljevic et al. 2023). In the meantime, the further spread of the species in Slovenia and Croatia has been documented. In Croatia, new discoveries have been made in the Peri-Panonian and Dinaric regions of the country, in very different habitat types: in rare sand dune vegetation, severely disturbed beech and fir managed forests and in peat bogs affected by desiccation (Šegota et al. 2021). In Slovenia, new records have been made in the alpine, subalpine and Dinaric phytogeographic regions of the country, in disturbed habitats and habitats with low human or animal impact. Although it has been naturalised in Slovenia, it is still not considered an invasive species. *C. introflexus* is generally considered an indicator of acidic substrates, with most records coming from such habitats (Kriebitzsch and Ewald 2011). However, in Slovenia, the majority of records are from carbonate bedrock, contrasting with the typical European ecological pattern (Strgulc Krajšek et al. 2023).

Amongst the invasive mosses worldwide, *C. introflexus* is probably the most minacious. This pioneer species is spreading rapidly, often threatening species-rich and regionally rare habitats (Klinck 2010). This is why the effects of this invasive species is the best studied amongst all invasive bryophyte species (Essl et al. 2014). Since the 1980s, researchers have studied the ecological characteristics of *C. introflexus* invasion into different ecosystems and its ability to compete (e.g. Berg 1985). Although this species with a wide ecological range can be found in a large number of European habitats, it is primarily a successful early invader in open spaces and gaps where vascular plants do not pose a threat (Żarnowiec et al. 2019). For example, *C. introflexus*, is a typical pioneer species in vacuum-harvested peatlands in Latvia (Priede and Mežaka 2016). In contrast to most alien bryophytes, which usually colonise human-disturbed environments (such as gardens, roadsides and walls), *C. introflexus* colonises semi-natural vegetation, including pine forests and plantations, (disrupted) bogs and coastal dunes (Essl and Lambdon 2009), affecting resident species composition (Essl et al. 2014). Bare, acidic soils favour the growth of *C. introflexus*, as there is little competition from vascular plants. However, a study from Hungary (Csiky et al. 2015) suggest that human activity is not necessary for colonisation, as animal disturbance or biological processes can often create such bare, acidic soil surfaces that are ideal for its colonisation.

Considering the small number of invasive bryophyte species globally, it is not surprising that their impact on native ecosystems and the economy has been poorly studied historically. Although there are few studies focusing on the negative impact of invasive bryophytes (Schirmel et al. 2011, Bourgeois et al. 2012, Schirmel and Buchholz 2013, Pehle and Schirmel 2015), much is still obscure and further research is necessary. The detrimental effects of *C. introflexus* invasion were originally noted for the coastal sand dunes of the Netherlands (Van der Meulen et al. 1987) where it is known to form incredibly dense turfs covering large areas (Sparrius and Kooijman 2011). According to Klinck (2010), it has the greatest negative impact precisely in the coastal sand dunes of Northern Europe, inland dunes and disturbed peat bogs. Its dense cushions could disrupt many vascular plants from germinating their seeds and replace native moss and lichen communities (Equihua and Usher 1993). Invaded native populations may become colonies of *C. introflexus* with significantly reduced abundance of herbaceous vascular plants as well as the diverse moss and lichen flora (Biermann and Daniels 1997).

By stabilising the soil, binding leaf litter, changing the rate of decay and creating microhabitats that affect the composition of microfaunal communities, *C. introflexus* can change the composition of the ecosystem and the functioning of dunes (Pehle and Schirmel 2015). These changes may negatively affect certain microfaunal communites, for example, spiders and ground beetles, which are sensitive to changes in vegetation structure (Schirmel et al. 2011, Schirmel and Buchholz 2013). However, responses in other taxa can be neutral or even positive depending on the species considered and ecological context (Pehle and Schirmel 2015). Nevertheless, its invasion alters ecosystem equilibrium and functioning, highlighting the complexity of invasion biology and the need for extensive, evidence-based research prior to making general conclusions. As *C. introflexus* poses a threat to native vegetation, most investigations in Western Europe have focused on sandy habitat types and associated nutrient-poor grasslands (Sparrius and Kooijman 2011). However, little is known about the biology and extent of the species' distribution across Europe.

*Campylopus introflexus* is still expanding its range in Europe (Hassel and Söderström 2005) - south-eastern Europe is an excellent illustration of a newly-occupied area in Europe where currently exist invasion of new habitats. Bearing in mind that the introduction of invasive species to new habitats, as well as spreading throughout the region, is inevitable, special focus should be given to early detection and quick response (Srivastava et al. 2019).

One of the tools that can help in the early detection of the spread and new localities of investigated invasive species are species distribution models (SDM). SDMs are widely used in various fields, including conservation ecology and biological invasions (Guisan and Zimmermann 2000, Townsend Peterson et al. 2007, Chen et al. 2020). SDMs correlate known distribution data of species with environmental variables to create a model that predicts species' ecological requirements and potential distribution over space and time (Kulhanek et al. 2011, Dyderski et al. 2017). For example, distribution modelling is widely used to predict the distribution of species in the future under the influence of climate change; that is, increase in temperature and CO_2_ levels. Specific eco-physiological and biological characteristics of bryophytes, including poikilohydry, sensitivity to moderately high temperatures and high dispersal ability make them ideal candidates for studying the effects of climate change.

In addition, invasion risk maps obtained by different models can serve for further early detection of spreading, as well as rapid response measures (Srivastava et al. 2019). Even though the use of such models leads to binary results on habitat suitability that may not be precise enough, the great advantage of these analyses is that they only require species occurrence data. This is particularly important considering that detailed population and distribution data are not available for some bryophyte species and/or regions. Distribution records of bryophyte species across Europe are markedly uneven, with some taxa and regions being more intensively explored than others. The region of south-eastern Europe is considered one of the bryologically less explored parts of Europe (Sabovljević et al. 2011). For example in Serbia, despite some old studies and species records, research has not been continuous and many parts of the country remain poorly investigated (Pantović and Sabovljević 2017). In addition to the history and continuity of research, species distribution data are significantly influenced by its traits such as size, ephemerality and taxonomic difficulties.

In this study, we investigated the potential current and future distribution of invasive moss species *C. introflexus* across Europe. The specific objectives of this research were to: (1) determine invasion risk map, that highlights potential geographic areas with suitable climate in the explored region, based on its previously known distribution in Europe; (2) to assess the impact of climate change on its potential suitable habitats and distribution in the future, as well as; (3) to define the climatic factors that have the greatest influence on its distribution in the investigated area.

## Material and methods

**Study Area and Species Occurrences**

For the modelling, occurrence records for Europe were obtained from the Global Biodiversity Information Facility database (GBIF.org 2025), supplemented with records from literature. The additional occurence records were obtained from systematic review of the more recent available literature on distribution of *C. introflexus* in Europe, which were not included in the GBIF source (see Suppl. material 1 for the full list of references used), like, for example, the presence of *C. introflexus* in Serbia (Sabovljevic et al. 2023). All occurrence data represented presence-only records. Before compiling the occurrence data, the R package *CoordinateCleaner* v.3.0.1 (Zizka et al. 2019) was used to pre-process and clean the GBIF data. After merging the cleaned GBIF and literature data, occurrence records were filtered using a grid approach with a resolution of 2.5 arcminutes, retaining only one record per grid cell. This resolution matches that of the ecological predictors and helps to reduce sampling bias and spatial autocorrelation (Boria et al. 2014).

**Environmental Variables**

An initial set of 19 bioclimatic variables at a spatial resolution of 2.5 arc minutes were obtained from WorldClim v.2.1 (Fick and Hijmans 2017) (see Suppl. material 2 for the full list of variables). Climate data from the Coupled Model Intercomparison Project Phase 6 (CMIP6) were used to assess the present and future potential distributions of *C. introflexus* (Fick and Hijmans 2017). The analyses included four future time periods (2021–2040, 2041–2060, 2061–2080, 2081–2100) and two shared socioeconomic pathways (SSP2-4.5 and SSP5-8.5), corresponding to projections of medium and high intensity radiative forcing. The scenarios were derived from the MPI-ESM1-2-HR global circulation model developed by the Max Planck Institute for Meteorology (Mauritsen et al. 2019).

The two scenarios were selected because they differ substantially from current climatic conditions and are associated with greater projected range changes. Moreover, it allows comparison with other species that have been studied under the most extreme scenarios. SSP2-4.5 and SSP5-8.5 are CMIP6 scenarios that differ in socioeconomic development, emissions and climate impacts. SSP2-4.5, an intermediate (*middle of the road*) scenario, assumes moderate population and economic growth, leading to emissions that peak mid-century and warming of approximately 2-3°C by 2100. Another scenario considered is SSP5-8.5, a high-emissions fossil-fuelled pathway that assumes rapid economic growth and continued reliance on fossil fuels, resulting in warming of approximately 4-5°C. Consequently, this scenario is associated with more severe impacts, including higher sea level rise, more frequent extremes and greater ecosystem stress.

To avoid multicollinearity amongst predictors prior to model development, variance inflation factors were calculated for the initial set of 19 bioclimatic variables using the R package *usdm v.2.1.6* (Dormann et al. 2012, Naimi et al. 2014). Variables with VIF values greater than 10 were excluded from further analysis, as this threshold is commonly used to indicate high levels of multicollinearity (Zuur et al. 2009). Retained variables included Annual Mean Temperature (BIO1), Mean Daily Temperature Range (BIO2), Temperature Seasonality (BIO4), Maximal Temperature of the Warmest Month (BIO5), Minimal Temperature of the Coldest Month (BIO6), Percipitation Seasonality (BIO15), Precipitation of the Wettest Quarter (BIO16) and Precipitation of the Warmest Quarter (BIO18).

**Modelling the Species Distribution**

Species distribution models were calibrated using a set of single algorithms, including Generalised Linear Models (GLM), Generalised Additive Models (GAM), Generalised Boosted Regression Models (GBM), Random Forest (RF) and Maximum Entropy (MAXNET) implemented in the *biomod2* v.4.2.4 R package (Thuiller et al. 2009). These five models were chosen to cover the advantages of each of them individually, both interpretability (GLM, GAM) and flexibility in covering more complex non-linear relationships (GBM, RF, MAXNET). Specifically, RF typically provides high predictive accuracy, while GLM offers greater interpretability, with GBM representing an intermediate level. Additionally, RF and GBM can automatically account for interactions amongst predictors, whereas in GLM, GAM and MAXNET, such interactions must be explicitly defined. During model development, no interaction terms were manually specified to avoid subjective selection, allowing machine-learning algorithms to capture interactions implicitly.

Given that only presence-only data were available, *biomod2* was used to generate pseudo-absences (Thuiller et al. 2009). Each algorithm was trained on three pseudo-absence datasets of 20,000 randomly selected points (approximately twice the number of occurrences), with five model runs per dataset. Evaluation was based on TSS and AUC, with only models exceeding the 0.7 TSS threshold included in the ensemble models (Allouche et al. 2006, Jiménez‐Valverde 2011).

Ensemble predictions were generated using two consensus approaches: committee averaging (EMca) and the ensemble median (EMmedian) in *biomod2* (Thuiller et al. 2009). These methods demonstrated the highest predictive performance according to ROC and TSS values and have been shown to improve predictive accuracy and stability compared to single-model approaches (Mohajane et al. 2021, Pantović et al. 2023). In order to gain a more comprehensive insight into model performance, both ensemble approaches were retained, since EMca emphasises agreement between models, while EMmedian emphasises overall predictions that are less affected by extreme predictions (Huang et al. 2024, Zhang et al. 2026).

Model outputs were categorised into three suitability classes, based on TSS-derived thresholds (Thuiller et al. 2009). Areas with EMca values greater than or equal to the TSS threshold of the EMca ensemble were classified as consensus-suitable; areas with EMmedian values between the TSS thresholds of the EMca and EMmedian ensembles were considered model-supported suitable; and areas with EMmedian values below the EMmedian TSS threshold were designated as unsuitable.

Differences in suitability values and percentage changes in the occupied area amongst the three suitability classes, which were based on TSS-derived thresholds, as well as across all analysed future climate scenarios, were tested in R using the Kruskal–Wallis test followed by the Dunn post hoc test (R Core Team 2024).

## Results

After cleaning, merging and thinning the data, n = 12,385 occurrences of *C. introflexus* in Europe were retained for modelling its potential distribution (details of the occurence records used for modeling are provided inSuppl. material 4). All algorithms used in this study effectively predicted the presence of *C. introflexus* in Europe, with relatively similar ROC and TSS values. The mean ROC and TSS values across single-algorithm models were 0.979 ± 0.003 and 0.896 ± 0.009, respectively. Amongst individual models, RF achieved the highest ROC and TSS values, followed by GBM, GAM, GLM and MAXNET.

For the ensemble model, all individual models were included, as the TSS of each exceeded 0.7. The ensemble model, based on committee averaging, had ROC and TSS values of 0.991 and 0.917, while the ensemble model based on the median had ROC and TSS values of 0.983 and 0.892, respectively. These differences may result from better agreement amongst the individual models in EMca, rather than from greater predictive power compared to EMmedian or single-model approaches. This indicates a relatively low level of uncertainty amongst individual algorithms, as confirmed by the uniformly high model performance values (see Suppl. material 3 for detailed performance metrics).

**Figure 1.**
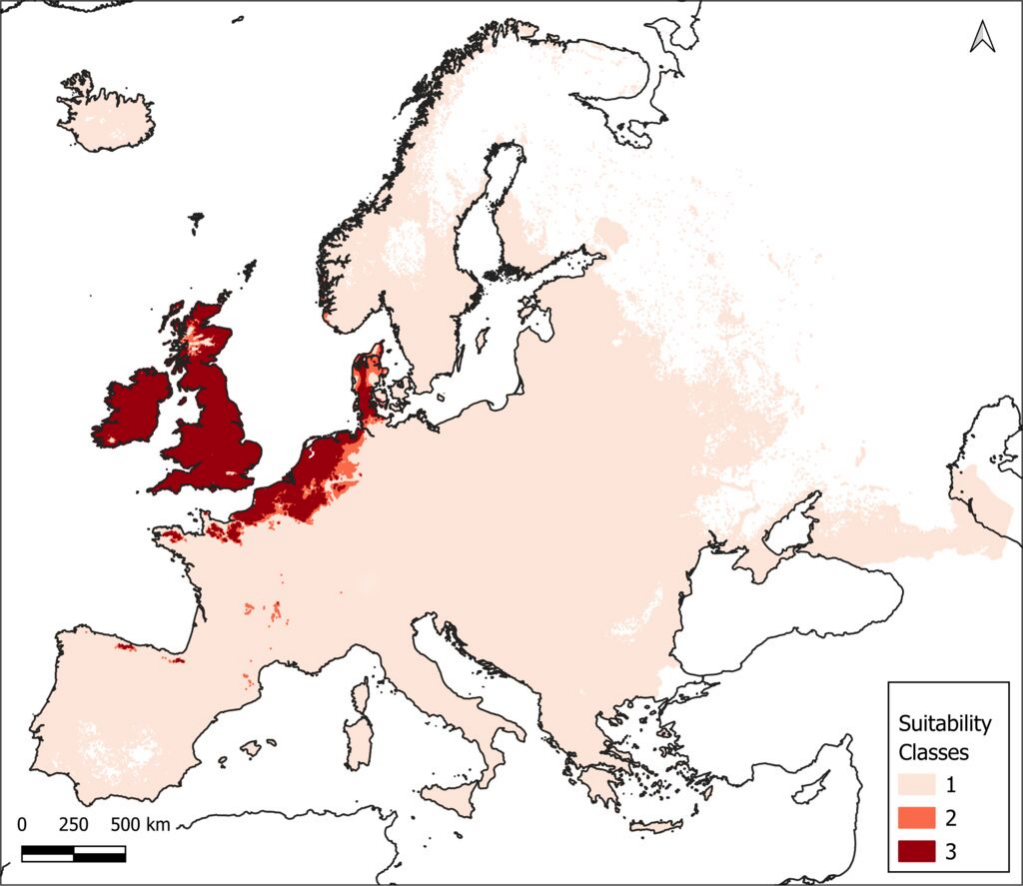
Map of Europe with indicated areas with suitable climate for *C. introflexus* as predicted under current climate conditions (1970–2000). Suitability classes: 1 - Unsuitable, 2 - Model-supported suitable and 3 - Consensus suitable.

The consensus-suitable habitat area for *C. introflexus*, as predicted by the EMca ensemble model and determined by applying the TSS threshold under current climate conditions (1970–2000), was 403,954.9 km² (7.39% of the total area). The model-supported suitable area, predicted by EMmedian values between the TSS thresholds of the EMca and EMmedian ensembles, was 77,459.1 km² (1.42% of the total area), while unsuitable regions covered 4,982,851.1 km² (91.19% of the total area) (Fig. 1).

**Figure 2.**
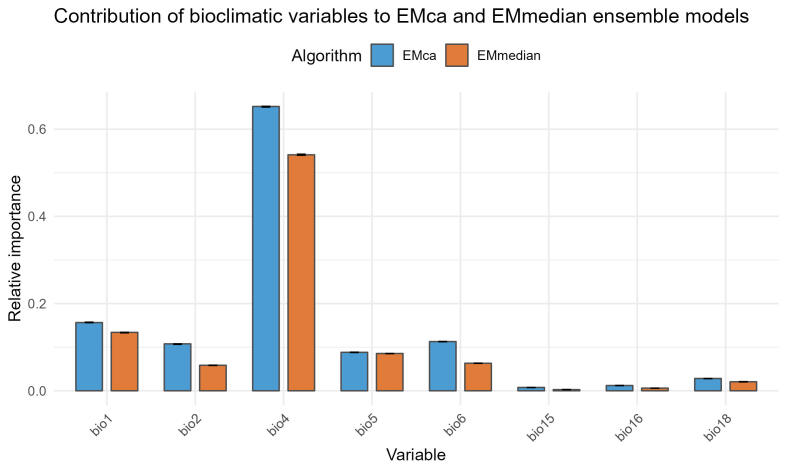
Relative importance of bioclimatic factors determining the potential distribution of *C. introflexus* in Europe. Abbreviations: BIO1-Annual Mean Temperature; BIO2-Mean Diurnal Range; BIO4-Temperature Seasonality; BIO5-Max Temperature of Warmest Month; BIO6-Min Temperature of Coldest Month; BIO15-Precipitation Seasonality; BIO16-Precipitation of Wettest Quarter; BIO18-Precipitation of Warmest Quarter.

Temperature Seasonality (BIO4) was by far the most important factor determining the potential distribution of the studied species amongst the eight predictor variables, when considering both ensemble approaches, with a mean relative importance of 0.651 ± 0.002 for EMca and 0.542 ± 0.002 for EMmedian (Fig. 2). Other temperature-related factors followed in importance, while the significance of precipitation was almost negligible. Specifically, in the EMca and EMmedian ensembles, the relative importance of the predictor variables was 0.157 ± 0.001 and 0.133 ± 0.001 for Annual Mean Temperature (BIO1), 0.107 ± 0.001 and 0.058 ± 0.001 for Mean Diurnal Range (BIO2), 0.088 ± 0.001 and 0.085 ± 0.0003 for Maximum Temperature of the Warmest Month (BIO5), 0.113 ± 0.001 and 0.063 ± 0.0001 for Minimum Temperature of the Coldest Month (BIO6), 0.008 ± 0.0001 and 0.003 ± 0.00003 for Precipitation Seasonality (BIO15), 0.012 ± 0.0002 and 0.006 ± 0.00007 for Precipitation of the Wettest Quarter (BIO16) and 0.028 ± 0.0001 and 0.021 ± 0.001 for Precipitation of the Warmest Quarter (BIO18), respectively.

Across all future scenarios, on average, consensus-suitable regions accounted for 2.70 ± 1.22% of the total area, model-supported suitable regions for 0.80 ± 0.13% and unsuitable regions for 96.51 ± 1.29% of the total area. These future patterns of suitability do not represent absolute habitat suitability, but rather relative suitability, based on agreement amongst ensemble models. According to the results of the Kruskal–Wallis test, the differences in future suitability levels amongst classes were significant (H = 19.0, df = 2, p < 0.001). Post hoc Dunn tests showed that the unsuitable class differed significantly from both the consensus-suitable (Z = 2.44, p < 0.05) and model-supported classes (Z = 4.34, p < 0.001), while there was no statistically significant difference between the last two classes (Z = −1.91, p > 0.05).

**Figure 3.**
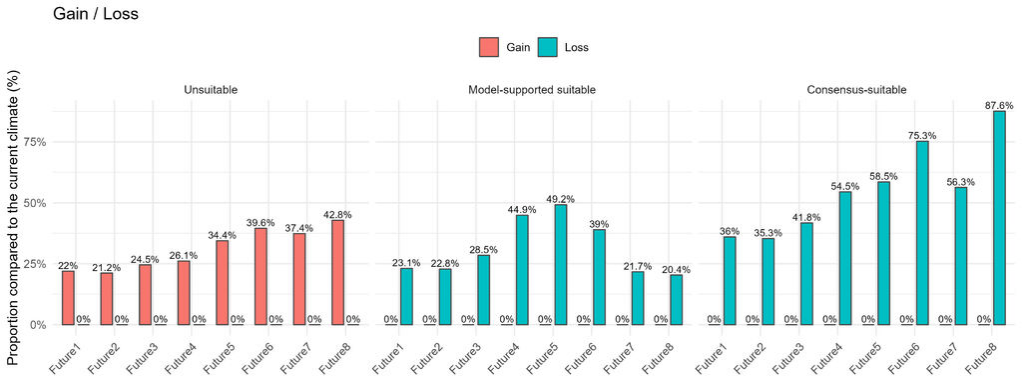
Changes in geographic areas with suitable climate under future climate scenarios - predicted percentage habitat gain or loss compared to the current climate. Abbreviations: Future1 - SSP2–4.5(21-40); Future2 – SSP5–8.5(21-40); Future3 - SSP2–4.5(41-60); Future4 – SSP5–8.5(41-60); Future5 - SSP2–4.5(61-80); Future6 – SSP5–8.5(61-80); Future7 - SSP2–4.5(81-100); Future8 – SSP5–8.5(81-100).

A general reduction of suitable regions for *C. introflexus*, including both consensus-suitable and model-supported suitable areas, was detected under all analysed future scenarios, while unsuitable habitats increased (Fig. 3). Specifically, compared to the current climate, consensus-suitable areas decreased by −31.19 ± 11.5%, model-supported suitable areas by −55.67 ± 18.5% and unsuitable areas increased by 31.00 ± 8.5%. Similarly to the case of future suitability levels, the results of the Kruskal–Wallis test showed significant statistical differences in percentage change in suitability amongst classes (H = 18.2, df = 2, p < 0.001). The results of the Dunn test were also similar: the unsuitable class differed significantly from both the consensus-suitable (Z = 4.26, p < 0.001) and model-supported classes (Z = 2.55, p < 0.05), while there was no statistically significant difference between the latter two classes (Z = 1.70, p > 0.05).

The Kruskal–Wallis test for each suitability class showed no statistically significant differences in percentage change across the analysed future climate scenarios (Consensus-suitable: H = 7, df = 7, p = 0.429; model-supported suitable: H = 7, df = 7, p = 0.429; unsuitable: H = 7, df = 7, p = 0.429).

**Figure 4.**
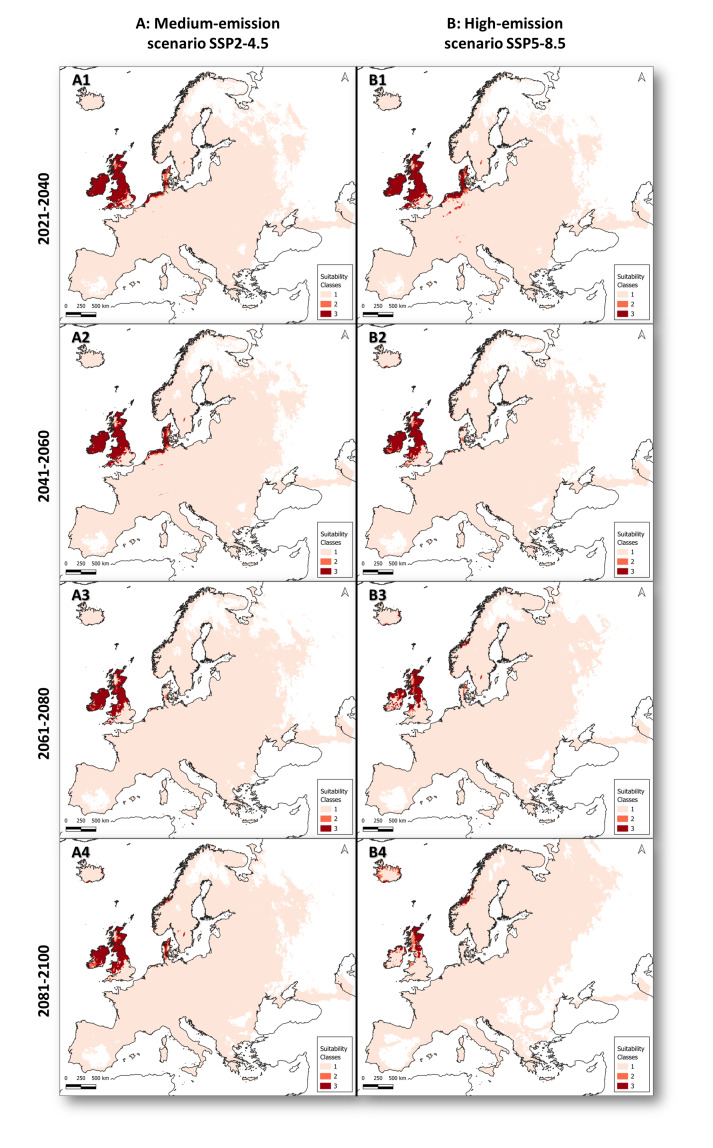
Predicted changes in the *C. introflexus* distribution in Europe under two climate scenarios: medium-emission scenario (A) and high-emission scenario SSP5–8.5 (B) across four future time periods (1: 2021-2040; 2: 2041-2060; 3: 2061-2080 and 4: 2081-2100), based on species distribution modelling. Suitability classes: 1 - Unsuitable, 2 - Model-supported suitable and 3 - Consensus suitable.

The analysis of range shifts indicated a substantial decrease in consensus-suitable areas, that is, geographic areas with highly suitable climate. Under the medium-emission scenario (SSP2–4.5), the consensus-suitable prediction range showed average declines of −36.02% for 2021–2040 (Fig. 4: A1), −41.75% for 2041–2060 (Fig. 4: A2), −58.55% for 2061–2080 (Fig. 4: A3) and −56.30% for 2081–2100 (Fig. 4: A4). Under the high-emission scenario (SSP5–8.5) for the same periods, a continuous decrease in the average area of suitable habitats was observed, from −35.33% in 2021–2040 (Fig. 4: B1), −54.50% in 2041–2060 (Fig. 4: B2), −75.27% in 2061–2080 (Fig. 4: B3), to −87.60% by 2081–2100 (Fig. 4: B4).

A decline in model-supported suitable areas, that is, moderately suitable climate, was also shown by the analysis of range shifts. The area reduction for model-supported suitable regions was slightly lower than for consensus-suitable habitat. Under the medium-emission scenario (SSP2–4.5), the consensus-supported suitable area prediction range showed average declines of −23.08% for 2021–2040 (Fig. 4: A1), −28.48% for 2041–2060 (Fig. 4: A2), −49.21% for 2061–2080 (Fig. 4: A3) and −21.68% for 2081–2100 (Fig. 4: A4). Under the high-emission scenario (SSP5–8.5) for the same periods, a continuous decrease in the average area with suitable climate was observed, from −22.83% in 2021–2040 (Fig. 4: B1), −44.89% in 2041–2060 (Fig. 4: B2), −38.98% in 2061–2080 (Fig. 4: B3), to −20.36% by 2081–2100 (Fig. 4: B4).

## Discussion

In this study, we modelled the current and potential distribution of the invasive moss *Campylopus introflexus* across Europe and assessed how climate change may affect its range, highlighting the key climatic factors shaping its distribution. The main findings of this study indicate that the invasive species *Campylopus introflexus* is likely to experience decline of areas with suitable climate across Europe under future climate scenarios. This projected decline is mainly driven by temperature seasonality (differences between the warmest and coldest periods of the year) and minimal temperature of the coldest month. These two climatic factors were identified as the variables that have the greatest influence on the current and future distribution of the examined species.

Bryophytes, generally, are highly sensitive to changes in temperature and moisture levels (Gignac 2001). As expected, previous studies have indicated that the expansion of *C. introflexus* was linked with climate that is similar to their native range and species did not show evidence for niche expansion in the invaded area (Mateo et al. 2014). Similarly, Essl et al. (2014) showed that climatic conditions, such as yearly mean precipitation, are an important predictor for the naturalisation of alien bryophytes.

Suboceanic temperate species, such as *C. introflexus*, occur in temperate regions influenced by the oceanic climate. These areas have relatively low temperature seasonality compared to continental Europe and are, therefore, characterised by mild winters, cool summers and high rainfall and humidity. Suboceanic temperate species are particularly sensitive to extreme temperatures and fluctuations; they cannot tolerate prolonged periods of summer droughts or long, cold winters with little precipitation. Thus, temperature seasonality is an important factor limiting their distribution. They require **stable and mild conditions prevailing in areas with low temperature seasonality**. Across Europe, **temperature seasonality increases both northwards and with distance from the coast.** This is why these species are mostly **coastal, western and oceanic-influenced** and are rarely found far inland or in northern continental regions. Even small increase in temperature seasonality may limit its range. Thus, distribution of *C. introflexus* in the study area and its current eastern range limit could be determined by values of temperature seasonality.

Researchers have shown that climate change is likely to have positive effects on the growth of some plants, particularly invasive species in non-competitive environments (Dukes 2000, Meyerson and Mooney 2007). Likewise, results of one meta-study suggest that elevated temperature, elevated atmospheric CO_2_ concentrations and N deposition may further promote invasiveness of the invasive alien plant species, while drought might inhibit it (Liu et al. 2017). Even though most bryophytes are able to survive short to moderate periods of drought due to their poikilohydric adaptation (Proctor et al. 2007), their survival and reproduction are highly dependent on the external environment. Many species are very sensitive to elevated temperatures, as they rapidly lose water when temperatures rise and air relative humidity decreases. High temperatures, therefore, largely limit the photosynthesis of many bryophyte species, further restricting metabolic activity and significantly impairing species survival (He et al. 2016). This is especially important considering that *C. introflexus* is a suboceanic temperate species that grows in full light (h photophyte) and in moderately wet or dry conditions (Dierßen 2001, Blockeel et al. 2014).

Apart from the characteristics of the invaded region and the history of introduction, invasion success is strongly influenced by species-specific life-history traits, particularly dispersal ability and reproductive strategy. The invasive *C. introflexus* has spread rapidly in Europe, apparently by its relatively small spores (12-14 μm), which are predominately dispersed by the wind (Hassel and Söderström 2005). In some cases, this species takes up to ten years after establishment to reproduce sexually, develop sporophytes and produce spores. However, after establishment, the species spreads efficiently through asexual propagation, by fragmentation of leaves (Glime 2017). Rapid production and spread of asexual propagules thus contributes substantially to the highly invasive potential of bryophyte species.

The model projections of this study clearly indicate that, after the initial phase of expansion in Europe, areas with suitable climate for the *C. introflexus* are going to decrease to a large extent under predicted and accepted climate scenarios. This further suggests a reduction of the species’ range in Europe in the regions where climatic conditions are expected to become less favourable and this seems to be rather large part of species range. Declining areas with suitable climatic conditions may cause the loss of occupied sites, potentially leading to population fragmentation and reduction of genetic connectivity. However, its extinction from Europe naturally is not an expected event under tested models. This is consistent with a study by Désamoré et al. (2012), which showed that temperate bryophyte species will respond to climate change by expanding their range during ongoing climate change, but will lose their range in southern regions of Europe in the long term. One of the most significant known effects of climate change, particularly rising temperatures, on bryophytes is the alteration of their distribution patterns (Zanatta et al. 2020). As a result, many species will be forced to shift their ranges to higher altitudes and latitudes in search of suitable habitats or, in this case, towards the north-western parts of Europe.

However, it should be noted that invasive species are dynamic by nature and their invasion mechanisms are sometimes difficult to predict (Srivastava et al. 2019). Although these models identify areas with suitable climate and other environmental factors, it has been shown that invasive species can invade areas broader than predicted, including those with different climates from their native ranges (Cordier et al. 2020). In addition, the potential negative impacts on local habitats and biodiversity often become apparent only 20-40 years after the first detection of invasive species (Essl et al. 2013). Furthermore, active measures to suppress their spread and restore invaded habitats are very limited and almost non-existent for this species. For this reason, it is important to better understand the distribution of the most emblematic invasive moss species in Europe, *C. introflexus*, as well as its potential impact on native habitats and communities, especially in the area of current spread.

Although species distribution modelling is a valuable tool for nature management, the processes influencing the species distribution, establishment and spread are inherently complex and the model outputs should, therefore, be interpreted with caution. SDMs primarily rely on correlations between ocurrence records and environmental variables. As a result, they typically focus on climatic factors and do not reflect on biotic interactions such as competition, dispersal ability, land use or species-adaptive phenotypic plasticity. These factors can strongly influence establishment success, distribution patterns and range dynamics, particularly under changing climatic conditions. Additional uncertainties may be related to the accuracy and spatial bias of the species' finding data, as well as limitations associated with model selection, choice of predictor variables and use of future climate scenarios. Consequently, real distribution of the species may differ from model predictions. Thus, SDM-based predictions should be seen as a starting point for investigating species ecology, potential impacts on native species and habitats, monitoring range changes and development of management strategies.

According to the obtained results, *C. introflexus* does not pose an invasion risk and is unlikely to expand its range under future climate change scenarios. However, climate change may have severe negative effects on native bryophyte species (e.g. Abubakar et al. 2024, Abay and Gül 2025), particularly through prolonged environmental stress that can ultimately lead to (local) extinctions. In the case of alien bryophytes, it has been shown that the most important negative effects of invasion are competition and the change in composition of native species and communities (Essl et al. 2014). Invasive species often prefer already disturbed habitats where competition is low. If a species exhibits sufficient resilience to change, such conditions may facilitate the colonisation of invasive species to new habitats and its range expansion, despite ongoing climate change and limiting climatic factors. However, biotic interactions that could positively or negatively influence the current and future spread of *C. introflexus* in this region remain poorly understood and should be more in focus of further research.

Despite the fact that climate change may negatively affect the European distribution of the investigated species in the future, research should continue to focus on its current distribution, spread, invasiveness and negative impact on native habitats. Essl et al. (2014) showed that negative impact on biodiversity and socio-economy is often noted a few decades after the initial invasion, which raise the importance of such studies. This is especially significant taking into account that number, frequency and spatial distribution of alien bryophytes are rapidly increasing worldwide (Essl and Lambdon 2009. Once an invasive species becomes established and naturalised, it is challenging to eradicate. For example, *C. introflexus*, in particular, can form uniform, dense mats that are difficult to treat and make it impossible to restore the natural habitat. In a habitat restoration attempt in the Netherlands, even mechanical soil removal and herbicide applications proved unsuccessful. After a few years, this species once again dominated. That is why monitoring of further regional spread is necessary for possible early intervention, which has proven to be the only effective measure to protect invaded and damaged native habitats.

Overall, while SDMs provide significant insights into potential distribution of the species, their projections should be interpreted carefully and serve as a basis for further ecological research and conservation.

## Conclusions

Using species distribution modelling, this study assessed the invasion risk of *Campylopus introflexus* in Europe and evaluated how climate change may alter its spatial distribution. Climate trends and projections in Europe do not appear favourable for the further spread of this invasive moss. This is due to the decline in areas with suitable climate and to the species' sensitivity to prolonged periods of heat and drought. This highlights the role of climate as a limiting factor in invasion dynamics. Nevertheless, the species may still pose a threat to local bryophyte flora, vegetation and associated species, particularly given its high capacity for asexual spread under current conditions, alongside sexual reproduction. Thus, the species' distribution dynamics should remain a focus of future research as field-based distribution studies are essential to validate model predictions. While models rely on incomplete or biased occurrence records that may consequently underestimate the species' true climatic suitability, this study provides a valuable foundation for further investigation of *C. introflexus* invasion and dynamics in south-eastern Europe, helping to prioritise regions for monitoring and management efforts.

## Supplementary Material

FFC60EF7-FA2D-5D5D-8713-68A0D76C81DD 10.3897/BDJ.14.e177294.suppl1 Supplementary material 1 References on distribution of *C. introflexus* in Europe used in addition to GBIF database. Data type references Brief description List of references used for additional (new) occurence records for *C. introflexus* in Europe. File: oo_1457928.docx https://binary.pensoft.net/file/1457928 Abubakar I, Šinžar-Sekulić J, Sabovljević M, Pantović J

25016F52-0F87-5BD1-8254-5B0186C8E0AB 10.3897/BDJ.14.e177294.suppl2 Supplementary material 2 List of Bioclimatic Variables used in the study Data type list Brief description An initial set of 19 bioclimatic variables with indicated variables that were used for modelling the potential distribution of the studied species. File: oo_1533526.xlsx https://binary.pensoft.net/file/1533526 Abubakar I, Šinžar-Sekulić J, Sabovljević M, Pantović J

804EB825-3F83-5DC1-B7D6-5D91E816F103 10.3897/BDJ.14.e177294.suppl3 Supplementary material 3 Summary of Model Performance Metrics by Algorithm Data type table Brief description This supplement presents a comparative summary of model performance across different algorithms used in the study. File: oo_1533559.xlsx https://binary.pensoft.net/file/1533559 Abubakar I, Šinžar-Sekulić J, Sabovljević M, Pantović J

CE84226D-4ADC-5D54-A95C-B454EC3B69D1 10.3897/BDJ.14.e177294.suppl4 Supplementary material 4 Data repository Data type list Brief description Details of the occurrence records used for modelling the distribution of *Campylopus introflexus* in Europe. File: oo_1534399.csv https://binary.pensoft.net/file/1534399 Abubakar I, Šinžar-Sekulić J, Sabovljević M, Pantović J
