# Supplementary material for: The current distribution of an invasive moss species Campylopus introflexus (Leucobryaceae) and models of its future range dynamics in two contrasting climatic scenarios in Europe
Source: Biodivers Data J. 2026 May 19;14:e177294. doi: 10.3897/BDJ.14.e177294 (PMC13213383; doi:10.3897/BDJ.14.e177294)
Supplement: Supplementary material 1 — References on distribution of C. introflexus in Europe used in addition to GBIF database. [file bdj-14-e177294-s001.docx]

Alegro A, Šegota V, Papp B, Deme J, Kovács D, Purger D, Csiky J (2018) The invasive moss *Campylopus introflexus* (hedw.) Brid.(Bryophyta) spreads further into south-Eastern Europe. Cryptogamie, Bryologie 39(3): 331-341. [10.7872/cryb/v39.iss3.2018.331](DOI:%2010.7872/cryb/v39.iss3.2018.331)

Blockeel TL, Afridi HUR, Bakalin VA, Czernyadjeva IV, Eckstein J, Erzberger P, Frey W, Fuertes E, Abdullah Gilani S, Hedenas L et al. (2007). New national and regional bryophyte records, 16. Journal of bryology 29(3): 198-198. [10.1179/174328207X209203](DOI:%2010.1179/174328207X209203)

Blockeel TL, Bakalin VA, Bednarek-Ochyra H, Ochyra R, Buck WR, Choi S, Cykowska B, Erdag A, Erzberger P, Kirmaci M, et al. (2009) New national and regional bryophyte records, 20. Journal of bryology 31(1): 54-62. [10.1179/037366808X343711](DOI:%2010.1179/037366808X343711)

Csiky J, Kováts D, Deme J, Takács A, Óvári M, Molnár VA, Malatinszky A, Nagy J, Barina Z (2017) Taxonomical and chorological notes 4 (38–58). Studia botanica hungarica 48(1): 133-144. [10.17110/StudBot.2017.48.1.133](DOI:%2010.17110/StudBot.2017.48.1.133)

Ellis LT, Bednarek-Ochyra H, Ochyra R, Benjumea MJ, Saïs LV, Caparros R, Lara F, Mazimpaka V, Dulin MV, Garilleti R et al. (2013) New national and regional bryophyte records, 35. Journal of bryology 35(2): 129-139. [10.1179/1743282013Y.0000000049](DOI:%2010.1179/1743282013Y.0000000049)

Górski P, Rosadzinski S, Rusinska A, Pawlikowski P, Wilhelm M, Zubel R, Piwowarski B, Staniaszek-Kik M, Fojcik B, Wolkowycki D et al. (2015) New distributional data on bryophytes of Poland and Slovakia, 3. Steciana 19(3): 163-176. [10.12657/steciana.019.018](doi:10.12657/steciana.019.018)

Górski P, Smoczyk M, Rosadziński S, Staniaszek-Kik M, Klama H, Pawlikowski P, Wilhelm M, Topolska K, Romański M (2016) New distributional data on bryophytes of Poland and Slovakia, 7. Steciana 20(3): 117-127. [10.12657/steciana.020.014](doi:10.12657/steciana.020.014)

Krajšek SS, Trnkoczy A, Cimerman ŽL, & Dakskobler I. (2023) Tujerodna vrsta mahu *Campylopus introflexus* (Hedw.) Brid. v Sloveniji. Hladnikia 52: 29-45.

Ellis LT, Aleffi M, Bednarek-Ochyra H, Bakalin VA, Boiko M, Calleja JA, Fedosov VE, Ignatov MS, Ignatova EA, Garilleti R et al. (2017) New national and regional bryophyte records, 51. Journal of Bryology 39(2):177-190. [10.1080/03736687.2017.1298297](DOI:%2010.1080/03736687.2017.1298297)

Mikulášková E (2012) Biology, ecology and invasion characteristics of *Campylopus* *introflexus* in the Czech Republic. Doctoral thesis, Charles University in Prague, Faculty of Science Department of Botany, 151 pp.

Mišíková K, Dobiašová K (2014) Checklist of bryophytes of the Borská nížina Lowland (Slovakia). Acta Botanica Universitatis Comenianae 49*:* 19-29.

Priede A, Mežaka A (2016) Invasion of the alien moss *Campylopus introflexus* in cutaway peatlands. Herzogia, 29(1): 35-51. [10.13158/heia.29.1.2016.35](http://dx.doi.org/10.13158/heia.29.1.2016.35)

Sabovljević MS, Tomović G, Niketić M, Lazarević P, Lazarević M, Latinović J, Latinović N, Kabaš E, Djurovic SZ, Kutnar L et al. (2020) New records and noteworthy data of plants, algae and fungi in SE Europe and adjacent regions, 1. Botanica Serbica 44(1): 81-87. [10.2298/BOTSERB2001081S](https://doi.org/10.2298/BOTSERB2001081S)

Sabovljević MS, Tomović G, Taşkın H, Assyov B, Škondrić S, Perić R, Sabovljević AD, Dragićević S, Marković A, Knežević J et al. (2023) New records and noteworthy data of plants, algae and fungi in SE Europe and adjacent regions, 15. Botanica Serbica, 47(2): 361-374. [10.2298/BOTSERB2302361S](https://doi.org/10.2298/BOTSERB2302361S)

Šegota V, Alegro A, Samardžić M, Rebrina F, Brigić A (2021) Invasive moss *Campylopus introflexus* (Hedw.) Brid. continues to spread through Croatia. In *4. hrvatski simpozij o invazivnim vrstama= 4th Croatian Symposium on Invasive Species* (pp. 36-36).

Širka P, Turisová I, Petrášová A (2016) Bryophytes of Cu-mine heaps in the vicinity of Banská Bystrica (Central Slovakia). Annales Universitatis Paedagogicae Cracoviensis Studia Naturae 1: 24-41.

Širka P; Kubešová S, Mišíková K (2018) Bryophytes of spoil heaps rich in toxic metals in Central Slovakia. Thaiszia - Journal of Botany 28: 59-77. <http://www.bz.upjs.sk/thaiszia>

Szűcs P, Csiky J, Papp B (2014) Neofiton *Campylopus introflexus* (Hedw.) Brid. elterjedése Magyarországon. Kitaibbelia 19(2): 212-219.

Szűcs P (2018) New data on the distribution of *Campylopus introflexus* (Hedw.) Brid. in Hungary. Acta Biologica Plantarum Agriensis 6*:* 133-139. [10.21406/abpa.2018.6.133](DOI:%2010.21406/abpa.2018.6.133)

Zubel R, Fojcik B, Wolski G, Uzieblo A (2009) First localities of neophytic moss *Campylopus introflexus* (Hedw.) Brid. in the Lublin district. Annales Universitatis Mariae Curie-Sklodowska 64(2): 45. [10.2478/v10067-010-0013-x](DOI:%2010.2478/v10067-010-0013-x)
